# Editorial: Scaling the Turbulence Edifice

**DOI:** 10.1098/rsta.2021.0101

**Published:** 2022-03-07

**Authors:** Jérémie Bec, Giorgio Krstulovic, Takeshi Matsumoto, Samriddhi Sankar Ray, Dario Vincenzi

**Affiliations:** ^1^ Université Côte d’Azur, INRIA, CNRS, CEMEF, Sophia-Antipolis, France; ^2^ MINES ParisTech, PSL Research University, CNRS, CEMEF, Sophia-Antipolis, France; ^3^ Université Côte d’Azur, Observatoire de la Côte d’Azur, CNRS, Laboratoire Lagrange, Boulevard de l’Observatoire CS 34229, NICE Cedex 4 06304, France; ^4^ Division of Physics and Astronomy, Graduate School of Science, Kyoto University, Kitashirakawa Oiwaketyo Sakyoku, Kyoto 606-8502, Japan; ^5^ International Centre for Theoretical Sciences, Tata Institute of Fundamental Research, Bangalore 560089, India; ^6^ Université Côte d’Azur, CNRS, LJAD, Nice, France

**Keywords:** turbulence, weak solutions, intermittency, scaling laws

## Abstract

Turbulence is unique in its appeal across physics, mathematics and engineering. And yet a microscopic theory, starting from the basic equations of hydrodynamics, still eludes us. In the last decade or so, new directions at the interface of physics and mathematics have emerged, which strengthens the hope of ‘solving’ one of the oldest problems in the natural sciences. This two-part theme issue unites these new directions on a common platform emphasizing the underlying complementarity of the physicists’ and the mathematicians’ approaches to a remarkably challenging problem.

This article is part of the theme issue ‘Scaling the turbulence edifice (part 1)’.

Turbulence is one of those deceptively simple phenomena that is encountered every day, at every scale, but whose comprehension continues to raise challenges to both modellers and theorists. For instance, the question of the regularity of solutions of the underlying equations that govern viscous flows is one of the holy grails of mathematics: It has been much searched after and discussed, but the answer has forever remained elusive. Indeed, ‘the turbulence problem’ stands like an unclimbed edifice in the inter-locking mountain-ranges of the mathematical and physical sciences. In this two-part, theme issue of the Philosophical Transactions of the Royal Society A, we present recent developments made at the front lines of physics and mathematics which provide a base, in the coming years, to scale this *turbulence edifice*.

Studies in turbulence have always straddled the boundaries between mathematics and physics. Historically, attempts to construct a unified picture from a blend of two distinct disciplines have been one of the instinctive enchantments of the subject. One may wonder what stands out in these recent developments which makes such attempts more urgent now. Let us take one example. Since Richardson’s [1] pioneering work a century ago, it has been understood that nonlinearities in turbulence generate successive small-scale fluid motions which are manifest in an energy cascade. The average behaviour of this cascade, predicted by Kolmogorov [2] in the 1940s, can now easily be checked by direct numerical simulations of the Navier–Stokes equations by using a standard computer equipped with, say, 20GB of memory. A long-standing central question, which stands within the remit of a statistical physicist, is how deviations from the average occur and what impact they have on the anomalous scaling laws of the velocity statistics. While many phenomenological models have rationalized the emergence of anomalous scaling, the underlying Navier–Stokes or Euler equations have, more often than not, little bearing on the genesis and development of such statistical theories. Recently, however, while resolving what is known as Onsager’s conjecture [3], mathematicians have also created a concrete model for such small-scale generation which is directly born from a weak (coarse-grained) formulation of the Euler equations, and which has a bearing on the origins of anomalous scaling. While conceptually and technically the physicists’ and mathematicians’ approaches are distinct, the commonality of the eventual goal is telling. This example suggests that there is an urgent need to break the *language* barrier between the two fields while emphasizing the underlying complementarity of these two approaches. Indeed, some recent advances in turbulence research have only strengthened the feeling that seemingly different approaches and results are connected at a deep and fundamental level.

This theme issue thus provides a *room* for an exchange of ideas between the two apparently parallel but fundamentally connected disciplines of the sciences. It also engages with a wider community which has looked at problems of turbulence, often in isolation. Equally enticing are some of the recent mathematical formulations that are amenable to the state-of-the-art numerical simulations that now lie at the physicists’ disposal. Similarly, recent developments in physics show promise for a fresh understanding of fundamental questions related to intermittency and broken scale-invariance. These are the important inputs that mathematicians seek when they attempt to construct weak solutions of the Euler equation which display tell-tale signatures of turbulence. Physicists are therefore now in a unique position to ally with mathematicians through state-of-the-art numerical simulations and fine-tuned experiments to uncover a wide range of singular solutions and probe emerging statistical properties. This is especially true with fine-scale measurements at large values of the Reynolds number where the bridge between Navier–Stokes and dissipative Euler solutions may not be as fanciful as was once believed. If we succeed, let us hope that we are on the cusp of a significant breakthrough in solving the turbulence problem.

One of the challenges in bridging the physical and mathematical approaches lies in the definition of the averaging procedure. Indeed, while the statistical theory of turbulence is based on the notion of ensembles averages, the existence of a stationary measure for the three-dimensional Navier–Stokes equations remains an open question. In the first paper of Part 1, De Lellis and Székelyhidi Jr. review how weak-convergence techniques and convex-integration methods can be combined to overcome this difficulty. The crucial role played by weak solutions in turbulence theory is also the topic of the review article of Mazzucato, where weak solutions are used to construct examples of the dissipative anomaly in passive-scalar transport. The phenomenon of the dissipative anomaly is then further investigated by Eyink, Kumar and Quan in the context of wall-bounded turbulence. In particular, regularization-group methods *à la* Onsager are used to derive a deterministic version of Prandtl’s relation between power-law scaling of wall friction and power-law profiles of the mean stream-wise velocity. When a flow is confined to a bounded domain the study of weak solutions requires a suitable weak formulation of the boundaries. This approach is presented in the article of Bardos and Titi, where regularity results for pressure in weak solutions of the Euler equations are proved in the case of a bounded domain with curved boundary.

Functional integration approaches to the statistical theory of turbulence are reviewed by Ohkitani. The focus is on the Hopf equation for the characteristic functional of the velocity field, and the Burgers equation is used to exemplify the techniques discussed in the article. It was mentioned above that, together with analytical approaches, numerical simulations play a fundamental role in the study of the statistical properties of turbulent flows. In their article, Buaria, Pumir and Bodenschatz use direct numerical simulations of high-Reynolds-number isotropic turbulence to examine the statistics of strain and investigate the physical mechanisms that cause its intense fluctuations. Gotoh and Yang consider an incompressible velocity field, a passive vector, and a passive scalar and study how in these systems the statistics of the gradient and the dissipation rate transitions from Gaussian to turbulent as the Reynolds number is increased.

Two articles are then devoted to intermittency. Dubrulle and Gibbon show how Sobolev estimates for weak solutions of the three-dimensional Navier–Stokes equations can be used to obtain rigorous bounds on the scaling exponents of the multifractal model of turbulence. Mailybaev & Thalabard identify a statistical scaling symmetry of the Navier–Stokes equations that holds in the inertial interval of a turbulent flow and restores the scale invariance broken by intermittency.

The existence of statistically conserved quantities and the associated scaling laws play a fundamental role in turbulence theory. Shavit, Vladimirova and Falkovich study the origin of the statistical scale invariance of the Kolmogorov multipliers in a family of models that connects wave-free incompressible flows to resonantly interacting waves. Panickacheril John, Donzis and Sreenivasan examine the laws of decay of kinetic energy for a variety of initial conditions and identify the necessary criteria for the observation of a power law. The article of Benzi, Castaldi, Toschi and Trampert concludes Part 1. It is shown that in a shell model of turbulence the temporal evolution of the kinetic energy consists of a phase of linear growth followed by abrupt avalanche-like energy drops. The scaling laws for the probability distribution of the time duration of the linear growth and the size of the energy drops are derived, and the relation between the two scaling exponents is discussed.

## The Uriel-esque approach and the Nice school of turbulence

Perhaps no one captures the spirit and soul of this issue more than our friend, collaborator and mentor, Uriel Frisch, who turned 80 in 2020. This issue is a tribute to his exceptional scientific career, with several seminal contributions in hydrodynamic and magnetohydrodynamic turbulence to his name, together with an enviable legacy of work which has ranged from the mathematical rigorous, the phenomenological, to pioneering the use of numerical simulations. Uriel was amongst the earliest to appreciate the beauty of small-scale intermittency, eventually leading to his introduction of the so-called multifractal formalism to explain turbulent fluctuations in the velocity and the energy dissipation, and its relation to real or complex singularities of the velocity field.

These went in parallel with studies of the dynamo effect and discoveries related to the central role played by helicity in transfers among scales. He was also among the first to explore the connection between the magnetic field and invariants (in a statistical sense) of turbulence. He made further contributions to the astrophysical community through his pioneering work on the cosmological reconstruction problem and the use of the Euler–Poisson equations.

Anticipating the power of scientific computing, Uriel has been strongly involved in the development of techniques for numerical simulation and their use in addressing fundamental issues in turbulence. Such uses and motivation led him to the invention of the efficiently-parallelized lattice-gas techniques which are guaranteed to converge to the Navier–Stokes equations. These were the precursor of Lattice–Boltzmann methods that are now widely used in both academia and industry to simulate not only idealized settings but also realistic situations with non-trivial boundary conditions.

Much less tangible, but equally important, is Uriel’s lasting contribution to creating a genuinely novel hub—the Nice school of turbulence—that embodies his truly unique approach. A master craftsman of young apprentices, Uriel has served as a bridge across the generations and genres by making his beloved Observatoire de la Côte d’Azur one of the most important ports of call and melting pots for both the beginner and the expert in turbulence research. Central to the fascination which many of us share for the Nice school is Uriel’s unique intellect, which seamlessly blends in discussions in history, linguistics, politics, literature, culture and the arts while figuring out complex questions of physics and mathematics. This engagement at so many levels is perhaps one of the many legacies of the Nice school of turbulence and its principal architect, Uriel.

This spirit is best captured in his widely-read text [4] which evolved from a set of lectures for first-year graduate students. Legend has it that his lectures given in a meeting room, now called Salle Michel Hénon in the Observatory of Nice, was always full of students with some sitting in the corridor outside. The appeal of these lectures was not just his energetic and entertaining style but his distinctive use of language in putting complicated matters into words without the use of equations or figures. Those who attended the lectures must have been delighted by this approach, but his PhD students and colleagues have subsequently suffered much from being asked to follow this practice whenever discussions with Uriel become serious.

Perhaps this style is a key element of Uriel’s mastery of erasure of the boundaries between physics and mathematics when it comes to turbulence. There seems to be another key element: Audacity. This is instrumental in asking a bold question or sometimes ‘so what?’ to assimilate knowledge of a different field. Uriel’s audacity is frequently unleashed during seminars given by his own guests. It then ends up in searching together with the audience for a better explanation, in words comprehensible for both sides.

May he proceed along his quest and remain the *conscience-keeper* and dissident of our field for years to come.

## Data accessibility

This article does not contain any additional data.
